# Amish fertility in the United States: Comparative evidence from the American Community Survey and Amish population registries

**DOI:** 10.4054/demres.2025.52.26

**Published:** 2025-04-29

**Authors:** Lyman Stone, Cory Anderson, Stephanie Thiehoff

**Affiliations:** 1 McGill University, Montreal, Quebec, Canada.; 2 Pennsylvania State University, University Park, PA, USA.; 3 University of Southampton, Southampton, UK.

## Abstract

**BACKGROUND:**

Quantitative studies of Amish population dynamics have been methodologically constrained by difficulties identifying Amish in national surveys. If Amish could be reliably identified in, for example, the American Community Survey (ACS), researchers could leverage its rich variables to document both demographic outcomes and their social predictors.

**OBJECTIVE:**

Cross-validate two methods for studying Amish populations by comparing fertility measures in the ACS with the Cross-sectional Amish Population and Environment Database-2010s (CAPED-2010s), a large administrative record database of North American Amish.

**METHODS:**

We identify potential Amish ACS respondents through combinations of the attributes (1) Pennsylvania Dutch language use, (2) absence of household telephone, and (3) farming. We then calculate fertility measures derived from both the CAPED data and ACS data samples (2000–2021). This comparative method allows us to assess whether the two samples produce demographic comparable estimates.

**RESULTS:**

Both methods produce remarkably consistent fertility statistics, including total fertility rates (just over six children), age-specific fertility rates (highest ages 20–29), and non-marital fertility (very low).

**CONCLUSIONS:**

The strong agreement between ACS- and CAPED-2010s-derived demographic estimates validates both approaches for studying Amish populations.

**CONTRIBUTION:**

The ACS’s rich social variables complement CAPED-2010s’ comprehensive demographic coverage, demonstrating the credibility of two separate large databases for studies of the Amish.

## 1. Introduction

The total fertility rate (TFR) in the United States has declined from slightly over 2 children per woman in 2007 to around 1.66 today, with causes not yet fully understood and the decline ongoing (see, e.g., Kearney, Levine, and Pardue 2022; Guzzo 2022; Guzzo and Hayford 2023). But have communities with historically very high fertility had the same fertility decline seen in more demographically mainstream communities? We focus on Amish populations, as Amish communities are largely integrated into many facets of American economic and social life, such as certain aspects of the market economy (Lutz 2017), modernized health care (Anderson and Potts 2020), and material culture (Tharp 2007), but famously not others, such as mass media/smartphones (Neriya Ben-Shahar 2021) and post-secondary education (Anderson 2015). We are not the first to identify the Amish as a credible control group to identify causes of change in American fertility (McKusick, Hostetler, and Egeland 1964): Bailey and Collins (2011) use Amish fertility trends to rebut certain arguments about US fertility during the baby boom.

The Amish are not strictly unique in their status as a high-fertility minority within low-fertility societies. Ultra-Orthodox Jews are another well-studied example (Stone 2023a). Prior research has also found that the Roma populations of Europe have maintained higher fertility rates, especially by keeping close within-group ties in relatively ethnically segregated communities (Battaglia, Chabé-Ferret, and Lebedinski 2021; Nestorová Dická 2021; Szabó et al. 2021). Within classical demography, Hutterite populations have been extensively studied as an example of high fertility rates persisting amid generally low-fertility societies. (Menken, Trussell, and Larsen 1986; Evans and Peller 2015).

Like other high-fertility groups, the Amish can theoretically receive converts from outside of their biological kinship groups (Anderson 2016), although such conversions are very few (Anderson and Thiehoff, under review; Scott 2007). Moreover, most high-fertility groups also generally practice degrees of shunning and isolation of kin who exit the religious confession, with the Amish particularly identified with this practice, although actual implementation varies widely across Amish affiliations (Petrovich 2017, 2023).

In this study, we leverage two data sources, comparing their results to improve understanding of Amish demography and to validate each approach. First, following Stone (2023a), we use a unique feature of the American Community Survey (ACS) and the social structure of Amish populations to identify Amish households for study. As discussed in more detail below, we identify Amish households through a mixture of language, nativity, and household appliances, a combination that allows us to produce relatively high-confidence estimates of Amish demographic behaviors. To our knowledge, the ACS is the only current regular survey in which Amish individuals are identifiable; surveys like the Current Population Survey and the National Survey of Family Growth do not ask about or provide data that would enable Amish households to be identified, and there is no longer a long-form census with relevant questions for identifying Amish households. This method builds on prior work, most notably Bailey and Collins (2011) but also Moledina et al. (2014), using language, nativity, and household appliances to identify Amish respondents in historic US decennial censuses.

Second, we present results from the Cross-sectional Amish Population and Environment Database-2010s (CAPED-2010s) (Anderson and Thiehoff, under review; Wasao, Anderson, and Mpody 2021). The CAPED-2010s includes data from 71 separate, independently produced North American Amish directories, or registries – printed books that include information about a select subset of Amish. The CAPED-2010s includes 54,731 Amish households, covering the vast majority of all Amish in North America. Remaining Amish populations, perhaps around 10% at the time of data collection, are not known to produce directories. This coverage gap includes many of the stricter denominations, such as Troyer-Stutzman and Swartzentruber Amish, as well as smaller Amish settlements in states without statewide directories, such as Ohio, Indiana, and Tennessee.

We adopt our dual approach for two reasons. First, pooling numerous Amish directories together to produce nationwide demographic estimates is a relatively new method, and the approach has not yet been externally validated. Nearly all Amish demography studies employ just a single directory (Colyer et al. 2017; Wasao, Anderson, and Mpody 2021). While this approach offers very high confidence that individuals identified are Amish, it is not prima facie clear that this approach will yield demographically reliable estimates or be representative of Amish populations generally. Second, survey-based approaches have been used for local Amish populations in the past (Enninger and Wandt 1982; Hewner 1998; Hurd 1985; Smith 1960), but they could be non-representative of Amish populations generally. By using both methods and comparing their estimates for key demographic processes related to fertility, we can establish whether or not these methods with different selectivity on unobservables yield similar results, suggesting good representativeness.

Numerous prior studies have assessed Amish fertility behaviors but usually in just one or a few Amish communities, and some are now decades out of date and cannot reflect any possible recent changes. For example, Ericksen et al. (1979) provides a fairly comprehensive study of Amish fertility but is now many decades out of date. Dorsten (1999) studies Amish marriages from 1884 to 1973, while Greksa (2002) computes total fertility rates from 1909 to 1967 but for just a single Amish community. Wasao, Anderson, and Mpody 2021 is fairly unique in presenting estimates for periods as recent as 2015 but again is able to analyze just one Amish community. Our study leverages nationwide survey data drawn from a sampling frame including all Amish communities, as well as a nationwide compilation of directories from almost all Amish communities.

## 2. Data and methods

Administered since 2000, the American Community Survey recruits a sample of approximately 3 million respondents per year for an extensive questionnaire, which includes key variables for this study: the language usually spoken at home, whether the respondent had a child in the prior year, and their age, sex, and marital status. After ACS respondents write in their language of home usage, US Census Bureau officials recode these open responses into fixed categories, including separate identification of German, Swiss, Pennsylvania Dutch, and Dutch. Pennsylvania Dutch (PD) is the usual label given in English to the variant of German (Deutsch, which is in fact not a variant of Dutch) spoken by most Amish communities. Pennsylvania Dutch speakers are very likely to be Amish (Anderson 2019), although they could also belong to one of several much smaller and historically related religious groups (Enninger 2002; Brown 2019; DeHaven 2010; Huffines 1986; Louden 2016) or might be genealogically related to Amish (they might be ex-Amish, for example) and live in Pennsylvania Dutch–dominant areas, such as Holmes County, Ohio. In general, these groups are all closely related geographically, denominationally, ethnically, or genetically to Amish. However, when ACS respondents give a language response, it is a write-in option, meaning respondents can write various things. Respondents might write in “Amish” or “German” or “Dutch,” depending on their exact Amish sect, their degree of familiarity with English linguistic nomenclature, and their own choice of self-representation. (In fact, in the ACS, those of Pennsylvania German ancestry report higher rates of speaking German, Dutch, Swiss, and other Germanic languages than even those of German ancestry.) As a result, some Amish people may be misclassified into other language groups.

To provide additional strength identifying Amish households, we also use variables related to household characteristics, specifically farm status and the presence of a phone in the household, essentially the same approach used with historic US Census data by Bailey and Collins (2011). Bailey and Collins (2011) found Amish fertility rates of approximately six children per woman for birth cohorts of women in the 1950s, similar to the rates we estimate, suggesting little secular change in fertility for Amish women between the 1970s and 2000s. Because many Amish groups restrict the use of phones, absence of a phone may strengthen the likelihood that a German-speaking household is Amish, for example. Furthermore, because Amish households are much more likely than American households generally to own and operate farms of various sizes, we consider farm status as well. In all figures, we present three different ACS-derived methods of identifying Amish households: individuals whose primary home language is Pennsylvania Dutch; individuals whose primary home language is Pennsylvania Dutch, German, Dutch, or Swiss and who live in homes without phones; and Pennsylvania Dutch speakers on farms without phones, a very restricted sample that is likely to be virtually 100% Amish survey respondents and plausibly very conservative Amish.

The CAPED-2010s, our non-survey-based sample, was compiled from 71 Amish-produced population directories. Directories are compiled by Amish, primarily for Amish, but can be acquired commercially or through direct contacts. Directories list household information within congregations, with all but the smallest directories including multiple congregations. For our purposes, the most important data fields from directories are birth dates and marriage dates with genealogical linkages of household members to other households.

CAPED-2010s is a repeated collection of life history data. In various years, communities assemble directories by asking households questions about marital status and marriage dates, number of children and their ages, and other social facts. Many individuals can be matched across multiple observations, though some communities have few directory editions or none at all. But even in communities where directory reporting is irregular, reasonable inferences about vital rates can be made for several years in the past through women’s fertility histories. Descriptive statistics for both ACS and CAPED samples are shown in Table 1.

## 3. Amish fertility indicators

**Figure 1:**
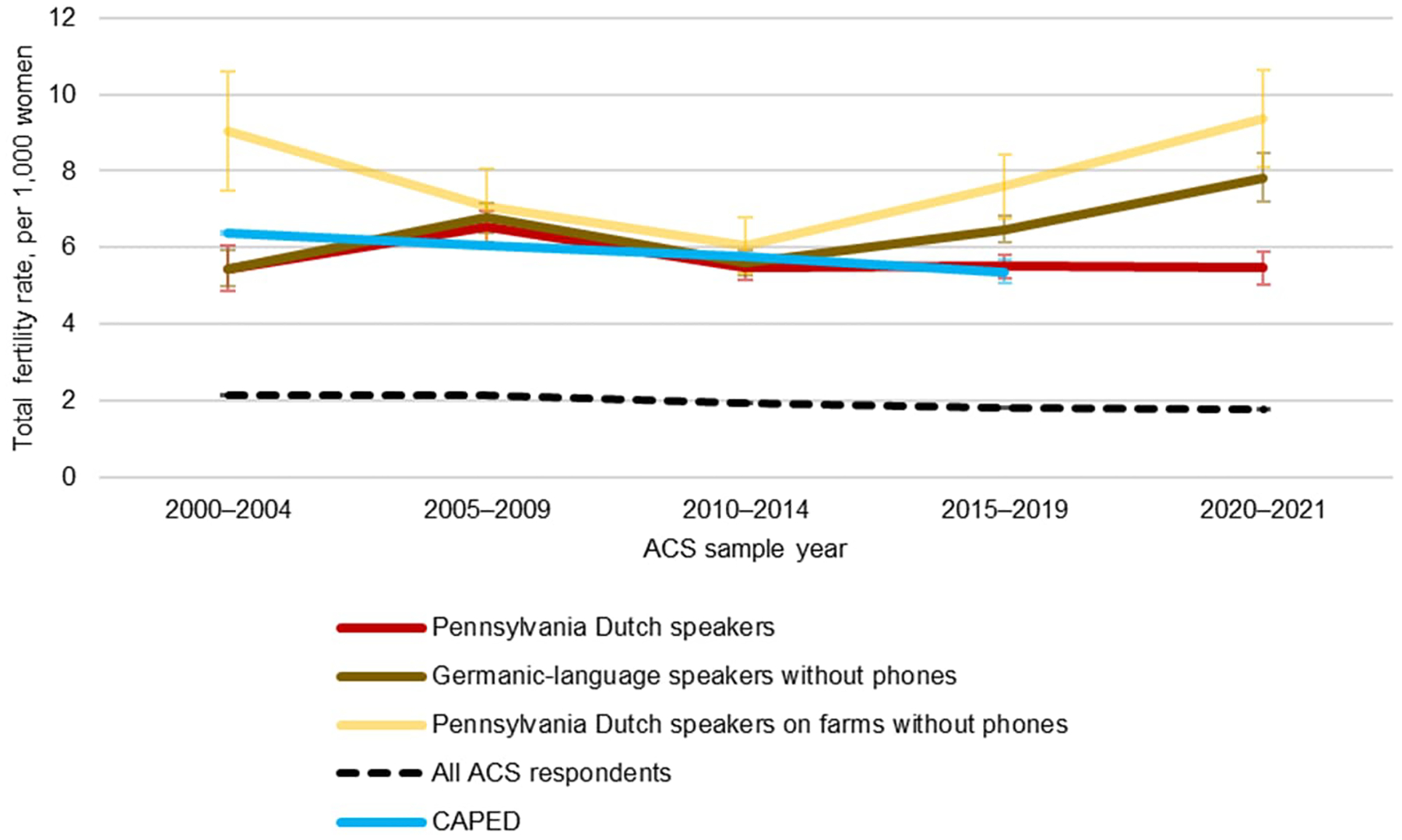
Total fertility rates by year and language spoken at home, ACS 2000–2021 and CAPED 2000–2019, 95% C.I.

Figure 1 shows estimates of annual (period) total fertility rates for Amish populations. For the Pennsylvania Dutch–speaker subsample (PD sample), recorded fertility rates are generally around six children per woman. Fertility rates appear to have risen significantly from 2000–2004 to 2005–2009 and then fallen through 2020–2021, suggesting some recent fertility decline among the PD sample.

On the other hand, for all Germanic-language speakers without phones, fertility rates have apparently risen over time, such that 2020–2021 birth rates are above 2000–2004 rates. Thus, in this subsample, fertility is rising. Finally, the estimates for Pennsylvania Dutch speakers living on farms without phones are less precise due to limited sample size, but they appear to show a U shape over time.

Fertility estimates from CAPED are, on the whole, very similar to ACS-based estimates for the PD sample. In general, the simplest sample definition, using all Pennsylvania Dutch speakers, yields the closest match to CAPED-estimated fertility. The other two samples may be higher, since by selection on nonuse of phones, they may capture stricter Amish sects; those stricter sects may also be slightly under-covered in CAPED. On the whole, however, the very close correspondence between CAPED and ACS estimates is encouraging, suggesting that they are both likely querying essentially the same underlying population.

**Figure 2:**
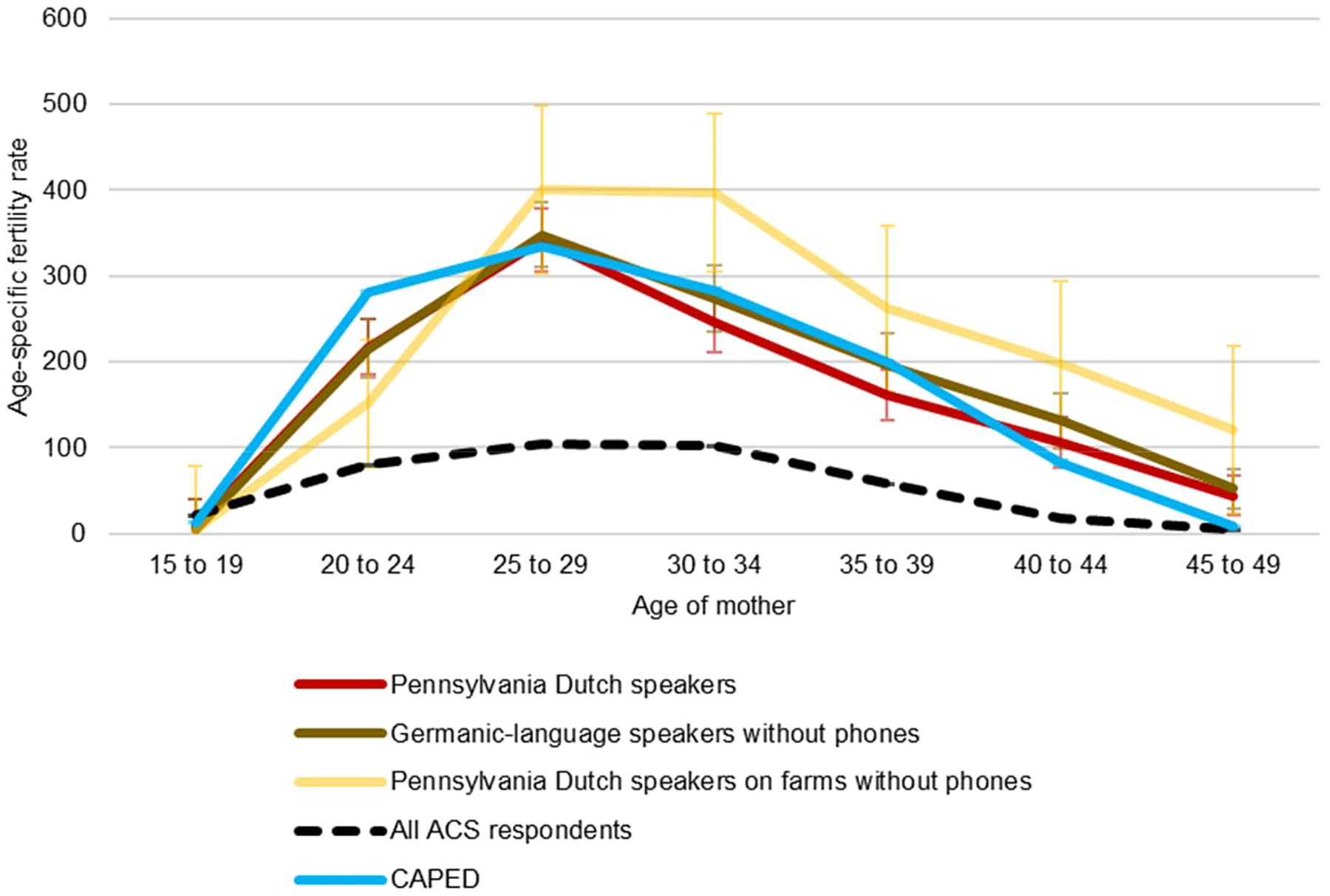
Age-specific fertility rates by age and language spoken at home, ACS 2000–2021 and CAPED 2000–2019, 95% C.I.

Pooling all years and respondents to ensure suitable sample sizes, Figure 2 shows estimated age-specific fertility rates (ASFRs) for all groups. Women in the PD “no phone on farm” sample have lower fertility in their early 20s than other women but higher fertility later in their reproductive lives. For all groups, estimates of teen fertility are actually lower than the average teen pregnancy rate in the ACS on the whole. This remains true in CAPED as well. Thus high Amish fertility rates do not seem to be driven by high teenage pregnancy among Amish women and girls. For ages 25 to 44, all four approaches to identifying ASFRs yield estimates with overlapping significance intervals, suggesting that they are not dramatically different, although the generally high estimates for Pennsylvania Dutch speakers on farms without phones do stand out. At ages 20 to 24, CAPED does show higher fertility estimates, with slightly lower estimates at ages 45–49.

Because both of our sources include data about women’s marital status, it is possible to estimate non-marital fertility proportions; only aggregated estimates across the whole period are shown due to small annual sample sizes of non-marital births. Among ACS respondents in general, 35.3% of reported births in the last year were to women who reported not being married. Some women may have married after a birth but before completing the ACS or may have exited a union in that period, thus this non-marital birth share is not a precise match for marital status at birth. Only 2% of births to Pennsylvania Dutch–speaking women were non-marital, with even lower figures for the other ACS samples. The figure from CAPED, where more exact marriage and birth dates are known, is 0.4% of births. Non-marital birth shares are extremely low in these communities, likely explaining the low prevalence of teen births.

## 4. Marital fertility

**Figure 3:**
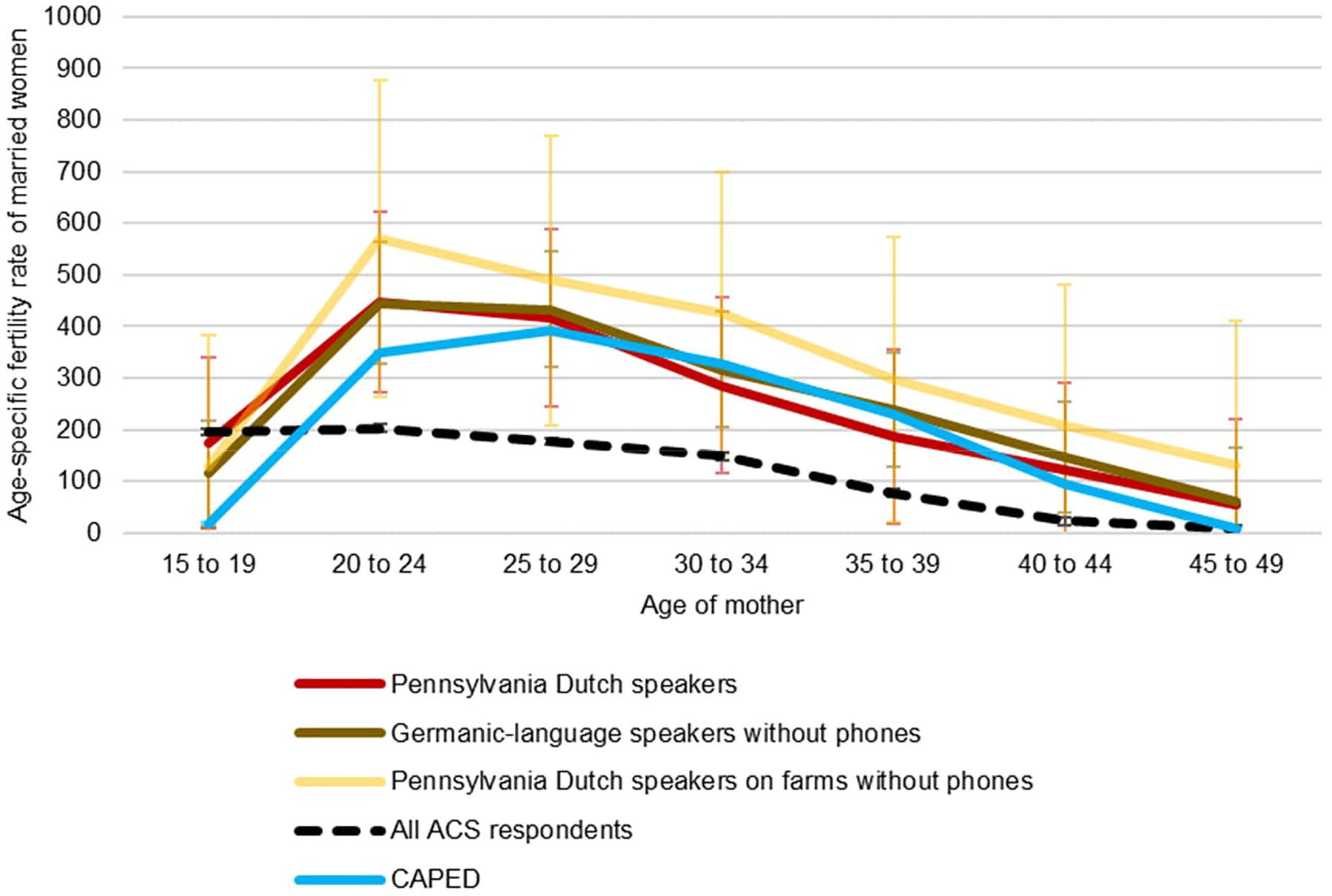
Age-specific marital fertility rates by age and language spoken at home, ACS 2000–2021 and CAPED 2000–2019, 95% C.I.

Next we turn to the topic of marital fertility. At least since Henry (1961) and especially since Coale and Trussell (1974), marital fertility rates have been of interest to demographers due to their proposed relationship to fertility control. We calculate age-specific fertility rates of married women to estimate marital fertility rates. These estimates can then be compared to historic “natural fertility” populations, and the standard Coale–Trussell *m* indicators can be calculated to assess any patterns of marital fertility control. The presence of such control would be interesting if it occurs, since Amish communities generally make limited use of contraception and, ostensibly, little to no use of clinical abortion (Anderson and Potts 2022). Figure 3 shows age-specific marital fertility rates.

Several facts stand out. First, ACS-respondent married teens have similar fertility rates, whether Amish or not. CAPED suggests very low fertility among married Amish teenagers. CAPED in general identifies lower marital fertility below age 25 and above age 39 than do ACS-based indicators. On the whole, whereas marital fertility rates for the US general population average 4.1, they are estimated at 7.1 in CAPED, 8.4 in the ACS for all Pennsylvania Dutch speakers, 8.8 for all Germanic-language speakers with no phone, and 11.2 for Pennsylvania Dutch speakers with no phone on farms. Thus elevated Amish fertility is not exclusively a product of higher marital exposure.

**Figure 4:**
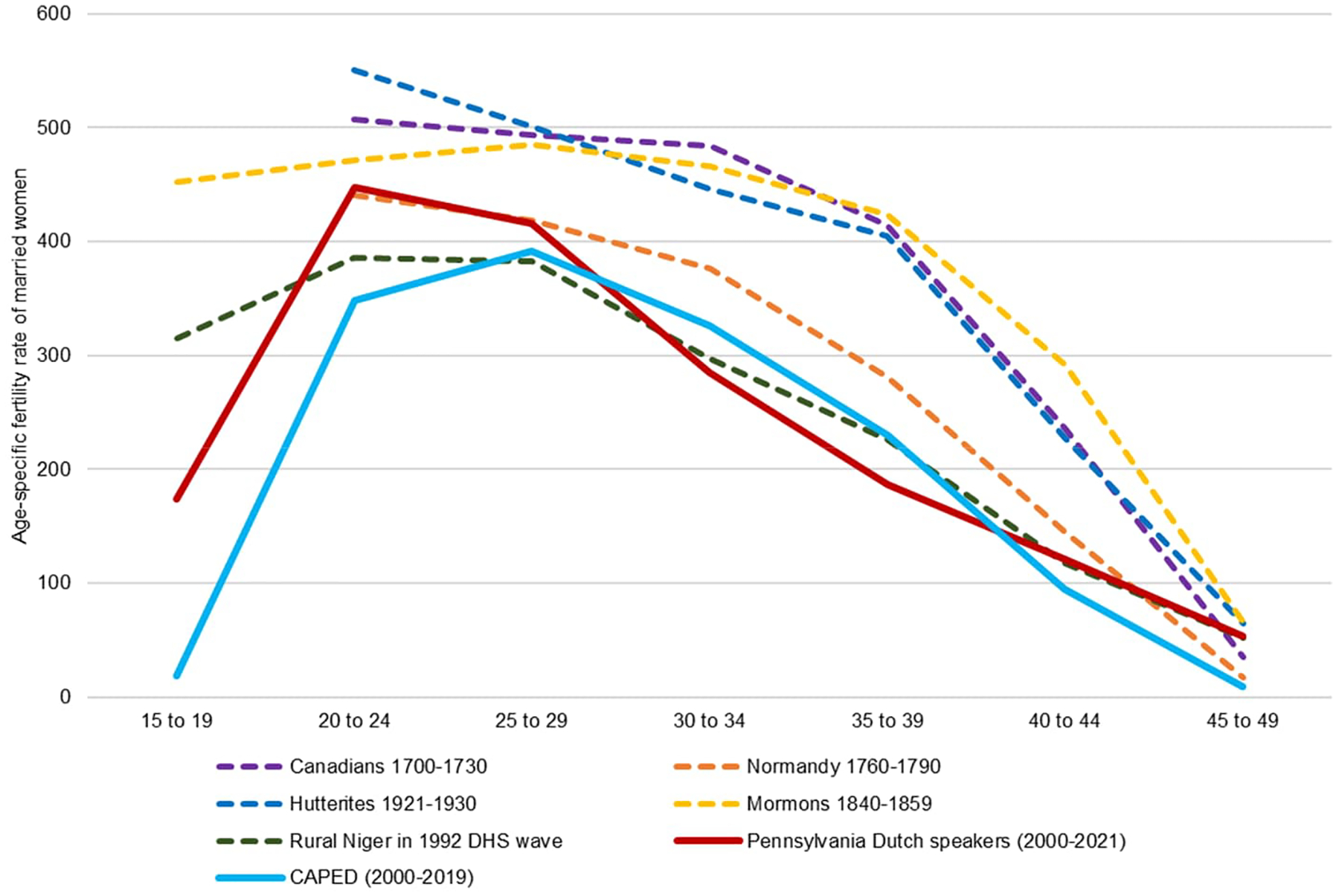
Amish age-specific marital fertility rates vs. natural fertility populations

However, while these rates are high, they may not necessarily indicate uncontrolled—or natural—fertility. Figure 4 shows how two of our Amish fertility indicators (Pennsylvania Dutch speakers and CAPED) compare to other natural fertility populations summarized in Stone (2023b). Amish married women age 20 and over have fertility rates comparable to some groups previously described as having natural fertility, such as rural women in Niger and, for Pennsylvania Dutch–speaking women in the ACS at some ages, Norman women in 1760–1790. But other classic examples of natural fertility, such as the Hutterites, French Canadians before 1730, and mid-19th-century Mormons, all exhibit much higher rates of marital fertility. This is suggestive evidence that Amish married women may use some forms of fertility control.

**Figure 5:**
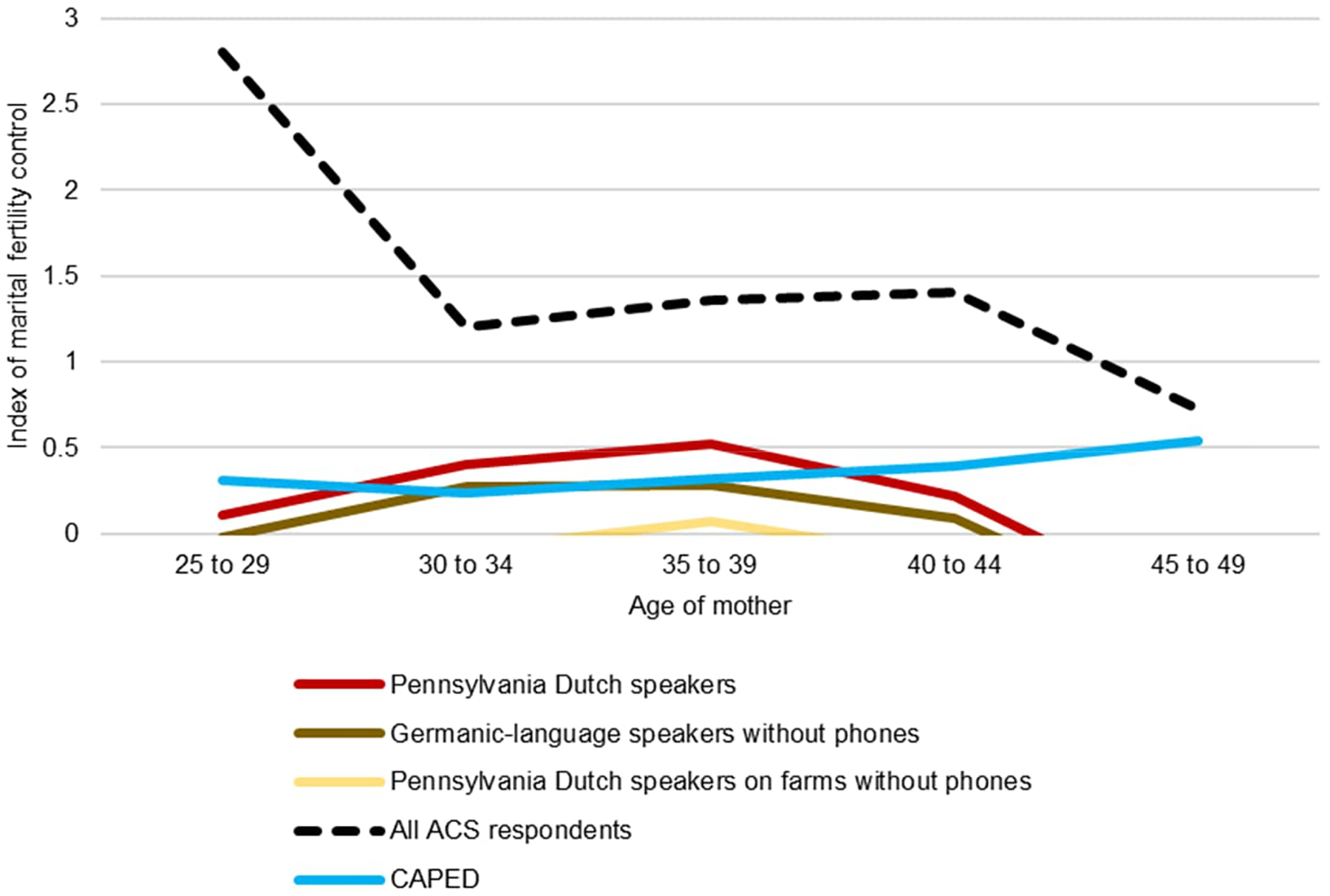
Coale–Trussell’s *m* for married Amish women

Finally, the classic indicator of age-specific reproductive control, Coale–Trussell’s *m*, can be calculated using both ACS and CAPED data. Coale and Trussell (1974) classically suggest that regimes with parity-specific reproductive control will exhibit stable values of *m* at values between 1 and 4, while natural fertility regimes will exhibit values near 0. We calculate Coale–Trussell *m* values for married Pennsylvania Dutch speakers in the ACS and for married women in CAPED (Figure 5).

General US women show strong evidence of reproductive control, while Amish women show far less. Pennsylvania Dutch speakers on farms without phones show essentially no evidence for control whatsoever. However, there is some modest evidence of possible reproductive control among all Pennsylvania Dutch speakers and in the CAPED sample. All CAPED age groups have values greater than 0, and women over age 44 may engage in some intentional stopping beyond natural fertility decline. Among Pennsylvania Dutch speakers, there is possible evidence of control behavior (whether stopping or spacing cannot be said from these data) around ages 35 to 39. On the whole, Amish women exhibit only very modest evidence of fertility control. The fact that the subsample indicative of the most traditionalism (Pennsylvania Dutch speakers without phones on farms) exhibited no evidence of control and has marital fertility rates around 11 children per woman likely suggests that the strictest Amish sects continue to experience natural fertility, while evidence for broader samples may suggest adoption of some forms of reproductive control among less-strict Amish denominations.

## 5. Conclusion

Most survey-based definitions of possible Amish respondents yield similar estimates of fertility over time and by age, and these estimates are themselves quite similar to those derived from administrative data compiled in CAPED. Despite different types of possible sampling errors, individuals identified as Amish using ACS language data and CAPED turn out to have highly similar demographic rates. One exception to this is on the question of marital fertility, where CAPED (which excludes some of the most traditional sects) and the broadest ACS sample show some modest evidence of fertility control while the most traditionalist ACS does not. Despite this variance on the technical question of fertility control within marriage, the cross-verification of more broadly construed marital and fertility behaviors serves to further elucidate the use and credibility of detailed ACS language and ancestry data for estimating demographic traits of small populations, as in Stone (2023a), while also demonstrating the nationwide credibility of the new CAPED, which has thus far not received extensive use.

As Amish fertility rates remain far above the US average and as the very modest pace of fertility decline (if any) promises to leave that large gap in place for some time to come, Amish fertility behaviors are likely to prove of enduring interest to demographers. In particular, the possible emergence of reproductive control within marriage among some less strict Amish denominations may be of particular interest. This study has used two different nationally representative samples of Amish to empirically support several findings that were already known from smaller-scale studies (Amish women average approximately six children each), as well as some that may be more surprising (Amish teen birth rates are not elevated; some Amish denominations may be experiencing the emergence of reproductive control). This study has focused on areas where CAPED and the ACS have essentially equivalent variables to demonstrate the credibility of both approaches. With that credibility demonstrated, we note that, with some additional database improvements, CAPED data could also be used to measure parity progression, intergenerational transmission of fertility, social status associated with religious leadership, denomination-specific fertility trends and traits, and other variables. Likewise, the ACS has a rich set of contextual and individual demographic variables that CAPED does not have and that may prove responsive to a variety of research topics of interest to Amish scholars (as in the case of Moledina et al. 2014). In the future, researchers can explore these questions confident of the general validity of the data for Amish populations.

## Figures and Tables

**Table 1:** Descriptive statistics

Age	Pennsylvania Dutch speakers		Germanic-language speakers without phones		Pennsylvania Dutch speakers on farms without phones		CAPED	
	*women*	*with a birth*	*women*	*with a birth*	*women*	*with a birth*	*women*	*with a birth*
15 to 19	1,944	19	2,044	15	465	3	12,657	964
20 to 24	1,510	326	1,565	331	244	34	10,147	2,271
25 to 29	1,325	475	1,346	477	212	83	8,688	2,774
30 to 34	1,191	343	1,169	363	225	88	7,094	1,991
35 to 39	1,053	210	919	202	171	48	6,016	1,206
40 to 44	910	106	773	115	153	35	4,996	460
45 to 49	755	35	732	45	149	15	4,038	55
	*men*		*men*		*men*		*men*	
15 to 19	2,082		2,138		485		13,514	
20 to 24	1,605		1,636		285		10,967	
25 to 29	1,406		1,351		200		8,766	
30 to 34	1,181		1,184		209		7,594	
35 to 39	1,046		933		174		6,092	
40 to 44	853		789		152		5,050	
45 to 49	756		730		144		4,017	
Phone availability								
No phone	7,696		17,309		3,268		15,462	
Has phone	9,876		N/A		N/A		13,998	
N/A or missing	45		N/A		N/A		80,176	
No phone share*	44%		100%		100%		52%	
Lives on farm								
No	11,073		10,363		N/A		62,910	
Yes	6,544		6,946		3,268		34,771	
N/A or missing	0				N/A		11,955	
Farm share	37%		40%		100%		36%	

* Includes 4,843 individuals with missing phone numbers as well as 75,333 individuals in directories without phone number data.
